# Digital recordings of a clinical encounter facilitate reflection in physical therapy students and clinicians

**DOI:** 10.3389/fmed.2024.1516724

**Published:** 2025-03-11

**Authors:** Anthony Kinney, Amy Nordon-Craft, Suzan Kardong-Edgren, Anshul Kumar, Anne Thompson

**Affiliations:** ^1^Physical Therapy Program, Department of Physical Medicine and Rehabilitation, University of Colorado Anschutz Medical Campus, Aurora, CO, United States; ^2^Department of Health Professions Education, School of Healthcare Leadership, MGH Institute of Health Professions, Boston, MA, United States

**Keywords:** digital recording, health professions education, physical therapy, standardized patients, simulation, reflection

## Abstract

**Background:**

Medical and health professions students use digital media in their educational pursuits. No studies have examined the process or utility of reviewing digital recordings of a clinical encounter. This pilot study examined how physical therapy students varied in their approach when compared to practicing physical therapists, in their self-reflection and assessment of a recorded physical therapist (PT) encounter with a standardized patient (SP).

**Methods:**

A single site, cross-sectional, mixed-methods design was used. Participants reviewed an 11-minute digital recording of a patient encounter and completed survey questions. Post-survey interview sessions were used to gain additional perspective from participants.

**Results:**

Ninety-two physical therapy students across three levels of training and twenty-seven physical therapists participated in the study. Self-ratings of perceived clinical ability increased with each year of training. First-year students (PY1) gave higher performance ratings to the PT than other groups. Seventy-five percent of respondents found the review of the digital recording to be a useful tool for reflection. A variety of approaches in the review process were found across groups, especially between clinicians and students.

**Conclusion:**

Review of a recording of a physical therapist’s encounter with an SP can be a useful educational tool for reflection across training levels of students and clinicians.

## Introduction

With the advent of handheld devices and online video sharing platforms, using prepared digital media to train medical and health science students has become easier and more acceptable. Access to digital recordings in education has resulted in students observing and analyzing video as a resource for learning. Increasing numbers of medical students are opting to watch recorded lectures of content at their convenience instead of attending in-person lectures (1). Previous research has found that medical students prefer the use of video recordings over reading materials (2). Medical educators in the 21st century need to be purposeful, proficient and intentional with incorporating video technology to develop future clinicians (3).

An example of intentional technology in medical education is the use of virtual patients to train clinicians. Virtual patients can be two-dimensional patients in a computer game or standardized patients (SPs). Virtual patients allow for the training and development of future clinicians by providing a standardized safe practice, and an on demand environment in which to learn (4). Video and virtual patients can prepare the developing clinician for challenging “real life” clinical encounters and developing reflective practice (5, 6). Scherer et al. (7) found that videotape review of trauma resuscitations resulted in behavioral change in team compared to verbal feedback alone.

Video is utilized in physical therapy education, but its impact on student development is not clear. Previous research has reported mixed results in using video to facilitate student physical therapists’ clinical development (8–10). The results differed as the previous studies used varying methodologies, including utilization of video and outcomes. A recent educational case study indicated that physical therapy students found benefits in reviewing their own Integrated Standardized Patient Examinations (ISPEs) (11). Exploring how students, at various semester levels in a program, view and analyze a recording of a physical therapist’s encounter with a standardized patient may lead to the development of a subsequent framework for guiding students viewing of recordings to maximize educational gains. The purpose of this study was twofold: (1) to determine whether watching a video of a physical therapist’s (PT) encounter with a standardized patient (SP) facilitated the viewer’s learning and self-reflection and (2) to explore how students at various educational levels viewed and analyzed a training video as compared to practicing clinicians.

## Methods

The IRB gave a certificate of exemption for this study. A mixed-method research design approach was used to allow for in-depth exploration and context of the subjects’ experience (12). Survey responses were collected, recorded, and managed online using REDCap (Research Electronic Data Capture) a secure, web-based software platform designed to support validated data capture for research studies (13, 14). In addition, participants were asked to participate in an optional post-experience individual interview or group sessions.

### Participants

Participants in the study were recruited from two groups. Graduate students enrolled in an entry level physical therapy education program were deemed “students” with the naming system based on professional year (PY1 for first-year students, PY2 for second year, PY3 for third year). Associated practicing clinical faculty who taught in the entry level PT program participated as PTs. Descriptive information of the student (PY) and clinician (PT) participants is presented in Tables 1, 2.

**Table 1:** Descriptive information of students (PY) reviewing recording.

Student year	Total number (number by gender)	Self-perceived ability
		0 = beginner 100 = expert
		Mean (±S.D.)
PY1	**33**	9.71 (±18.24)
	Female = 27	
	Male = 5	
	Agender = 1	
PY2	**33**	17.47 (±21.42)
	Female = 20	
	Male = 10	
	Agender = 2	
	Non-binary = 1	
PY3	**26**	25.62(±27.56)
	Female = 22	
	Male = 4	

**Table 2:** Descriptive information of physical therapists (PTs) reviewing recording.

Gender	Number	Years experience mean (+S.D.)	Self-perceived ability 0=Beginner; 100=Expert Mean (+S.D.)	Board certification	Fellowship trained	Practice setting (All that apply)
Female	17	12.54 (+10.17)	82.69 (+10.44)	10	4	8-Outpatient 4-Inpatient 4-Private Practice 1-Home Health 1-Other
Male	10	11.70 (+7.83)	77.70 (+16.87)	6	0	7-Outpatient 2-Private Practice 2-Other

### Clinical scenario and recording

**Figure 1:**
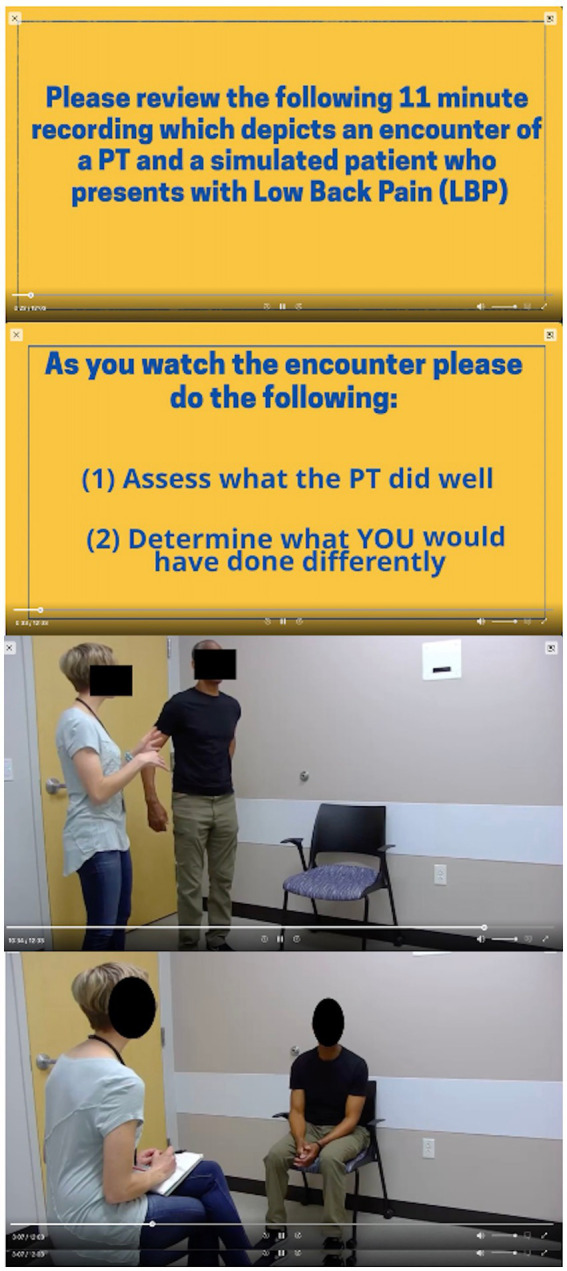
Screenshots from the digital recording of the physical therapist encounter with the standardized patient.

An 11-minute case video portraying a middle-aged man presenting to physical therapy under with reports of low back pain (LBP) and abdominal pain was videoed using best practice standards (15–17). The video was designed to highlight a common reason (LBP) for a patient to seek physical therapy care, and to rule out possible systems (gastrointestinal) involvement, through interviewing and physical exam. A script and written and verbal instructions were provided to both the PT and SP prior to the rehearsal of the scenario. A pilot test simulation of the scenario was conducted, and feedback was provided to the PT and SP prior to recording. The PT was instructed to perform at an intermediate level of PT skill throughout the encounter. A sequence of screenshots with guiding instructions and time stamps from the recording can be found in Figure 1.

### Survey design and distribution

A survey was developed which included demographic data, observation and debriefing questions (Appendix A). Two questions (#9 & #10) were based on the Plus-Delta approach to simulation debriefing (18, 19). After consenting to the study, participants immediately viewed the 11-minute video. Participants subsequently submitted proof of completion of video review then answered the survey questions. Survey responses were anonymous. Participants were also invited for post-study interviews with the secondary author. Interviews were conducted via Zoom, which is considered a viable resource for collecting qualitative data (20). A script (see Appendix B) was used for each interview session.

### Data collection

Deidentified data were exported from REDCap to an Excel file. Descriptive statistics, plots and linear and logistic regressions were performed in R (21). Narrative responses were downloaded and assigned a unique identifier for thematic review. NVivo, (Luminvero) was used to sort narrative responses. Transcripts were recorded from the interview sessions. The primary and secondary author independently reviewed narrative responses from the survey and interviews to prevent bias of results. Both authors independently generated codes and themes for the narrative responses from the survey and interviews, respectively. Both authors used a team-based, iterative approach to explore the respective data (22). All themes were developed by the reviewing, comparing, and analyzing codes. To ensure trustworthiness, the authors followed the constructs outlined in the Standards for Reporting Qualitative Research (23).

## Results

Out of 217 eligible students, 92 students participated (42.39%). The participation rate for clinicians was 26% (27 clinicians out of 104). Descriptive information of students (PYs) and clinicians (PTs) is found in Tables 1, 2. Students (PY) and clinicians (PT) provided self-ratings of their own perceived clinical ability (Tables 1, 2). The mean self-ratings increased with each year of training. The clinician self-ratings were closer to the expert rating, and higher for those who self-identified as female, rather than male.

**Figure 2:**
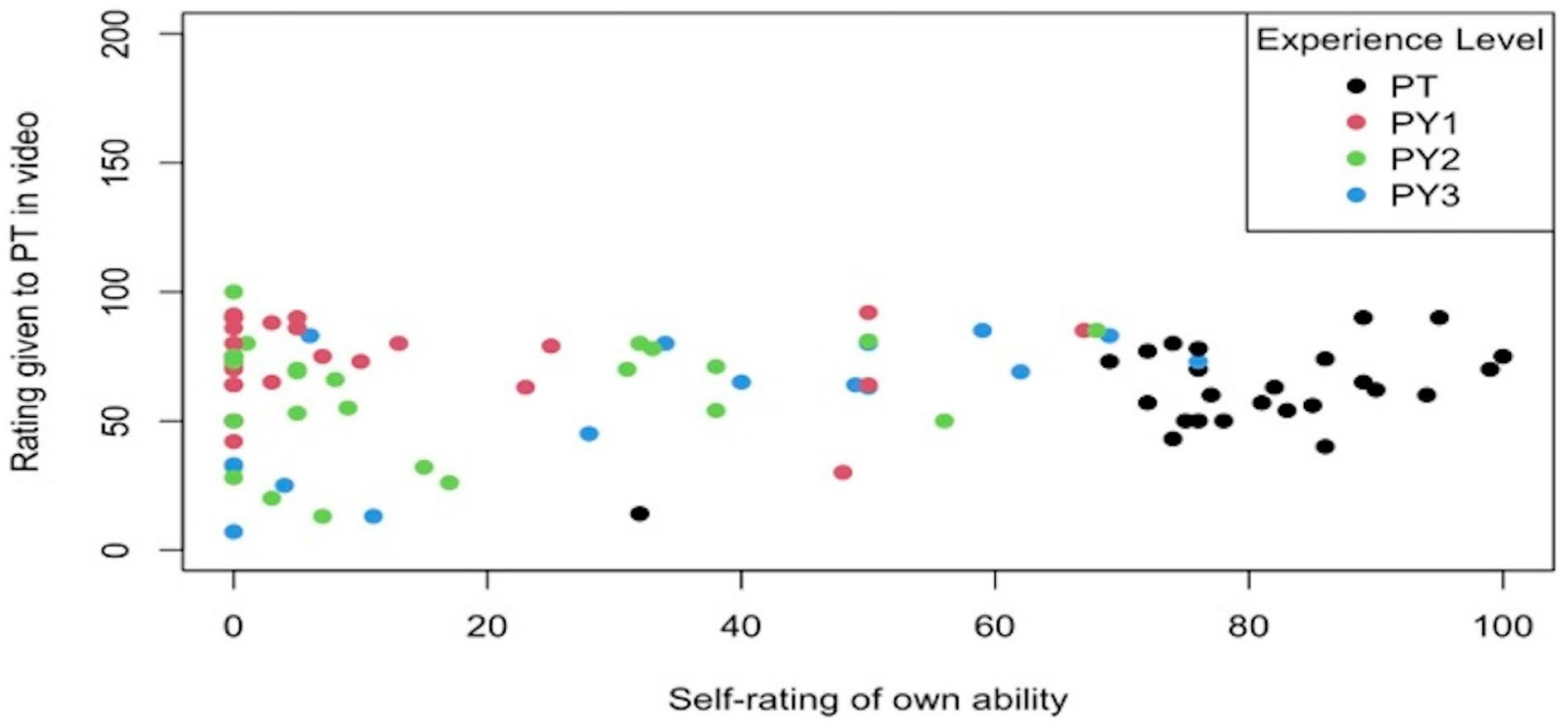
Scatterplot of rating of PT in recording with self-rating of perceived ability across level of experience. PY1, professional year 1; PY2, professional year 2; PY3, professional year 3; PT, licensed physical therapist.

The mean rating of the physical therapist’s performance rated by clinicians was 62.32 (± 16.69). First-year physical therapy students (PY1) rated the PT more highly than clinicians or other students. A comparison of participant’s own self-rating to the rating which they assigned to the PT in the recording is provided in Figure 2. Self-rating of ability in comparison to the rating of the PT in the recording is necessary to demonstrate context and engagement of the participant.

**Figure 3:**
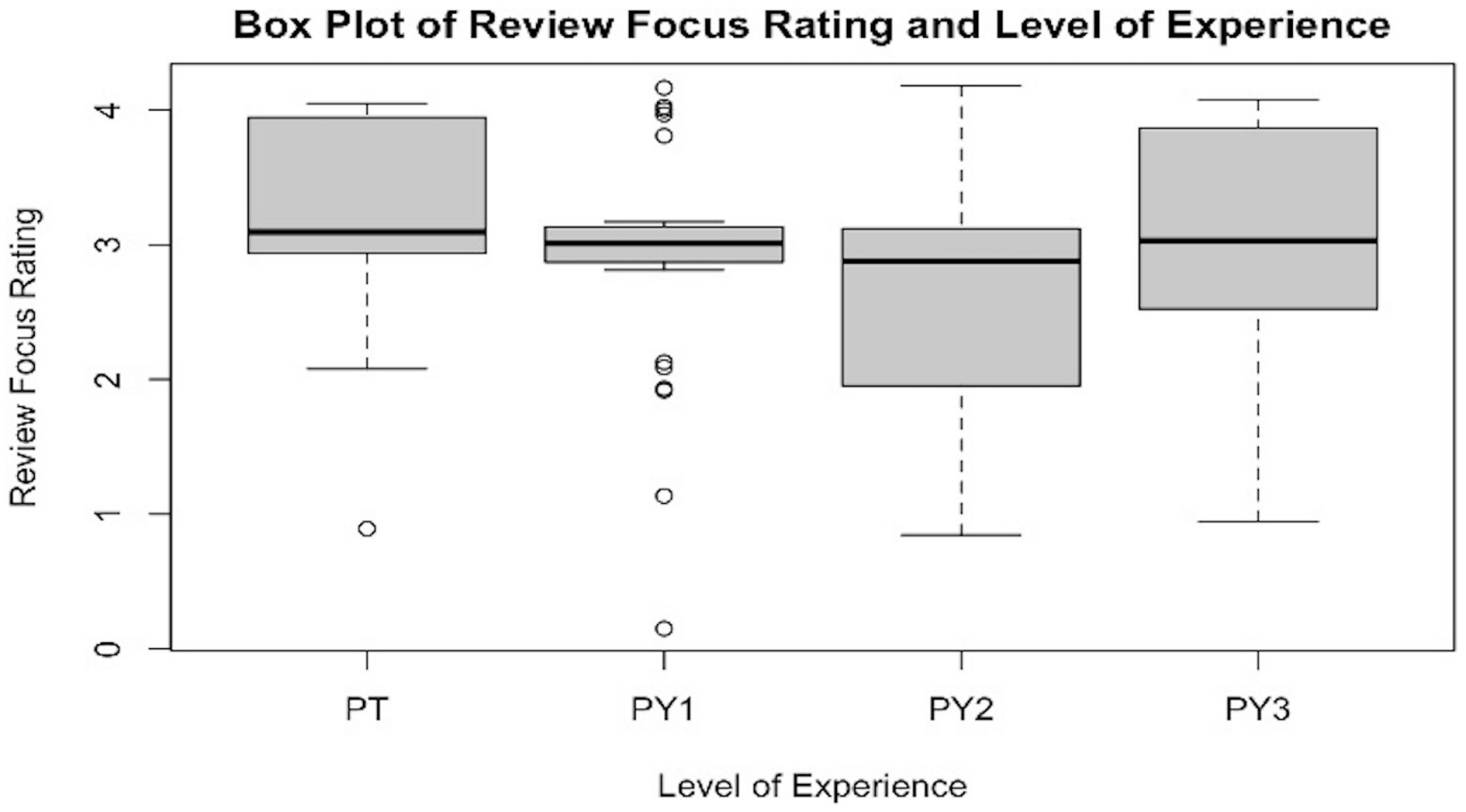
Boxplot of “review focus” and level of experience. Focus rating scale: 4 = strongly agree; 3 = agree; 2 = neutral; 1 = disagree; 0 = strongly disagree.

A summary of multi-tasking methods which students and clinicians used while reviewing the recording can be found in Table 3. Students across all levels of training appeared to use the fast play speed (e.g., 1.5x, 1.75x) setting while reviewing the recording, with PY3 students using it the most. Clinicians were less likely to use the fast play setting, although 25% of clinicians utilized the feature. The use of fast-forwarding through the recording was not used at all by clinicians (PT = 0%), but was by some students, mostly in PY2. Clinicians (PT) were more likely to take notes, whereas the method was much less utilized by all student groups. The pause feature was most utilized by PY3 students, with PTs utilizing it next most commonly. Clinicians (PT) had a higher mean rating of self-reported review focus as compared to all student groups, with 3rd year (PY3) students reporting the most focus of the three student groups (Figure 3).

**Table 3:** Self-reported multi-tasking while watching recording separated by experience.

Experience	Fast forward	Fast speed	Paused	Reviewed other websites	Text messaged	Took notes	Listened to music	Watched TV, movies and/or other videos	None of the above
PY 1	6%	51.52%	15.15%	0.00%	12.12%	9.09%	0.00%	0.00%	27.27%
PY 2	15.15%	51.52%	15.15%	3.03%	12.12%	9.09%	3.03%	0.00%	12.12%
PY 3	3.85%	53.85%	34.62%	7.69%	15.38%	15.38%	0.00%	0.00%	7.69%
PT	0%	25%	29.60%	11.11%	3.70%	40.70%	0.00%	0.00%	18.52%

PY1, professional year 1, PY2, professional year 2, PY3, professional year 3, PT, licensed physical therapist.

**Figure 4:**
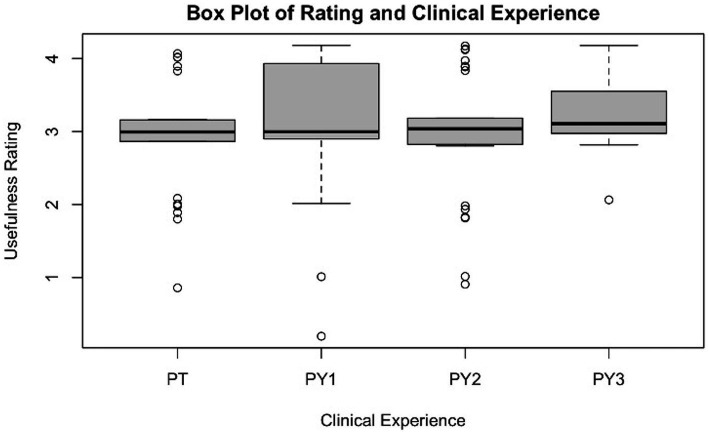
Boxplot of “review useful tool” and level of experience. Usefulness rating scale: 4 = strongly agree; 3 = agree; 2 = neutral; 1 = disagree; 0 = strongly disagree.

Overall, 74.79% of all respondents (89/119) answered either “Agree (3)” or “Strongly Agree (4)” when asked if reviewing the recording was “a useful tool in reflecting on my own professional ability and development.” Third year students (PY3) appeared to find the review of the recording most helpful in reflecting on professional ability and development, with a mean response of 3.22 (±0.52). PY1 students also agreed, with an mean response of 3.10 (± 0.94). PT and PY2 tended to fall just below “Agree,” with means of 2.92 (± 0.76) and 2.96 (± 0.82), respectively. A boxplot of responses separated by level of training can be seen in the in Figure 4. Two linear regression models were performed (see Appendix C). Regression 1 was used to examine whether the usefulness of digital recording was associated with year in training/experience as a clinician, controlling for demographic characteristics. No associations were found to be significant.

The second regression examined the relationship between level of focus in video review and year in training/experiences as a clinician. All students reported lower average levels of focus than licensed PTs, but only PY2s were significantly lower, by 0.69 points. There was no significant difference in levels of focus by gender.

On average, 1st year (PY1) students identified 2.25 (±1.24) distinct observations of actions or behaviors (items) that the physical therapist did well in the recording. Items that the PYI group tended to focus on more were about the approach and interaction with patient. The average gradually increased by level of training (PY2 = 2.41 ± 1.43, PY3 = 2.63 ± 1.52). Licensed physical therapists identified 3.58 (±1.70) items that the physical therapist did well in the recording. Example of items included comments concerning the PT’s skill with inquiry, explanation, screening, analysis, and assessment (Table 4).

**Table 4:** Counts, themes and example quotes from narrative responses to what the reviewer identified the PT in the recording doing well.

Level of experience	Item count for what the PT did well in the recording (Mean counts ± SD)	Themes	Example quote from narrative responses
PY 1	2.25 ± 1.24	Inquiry Explanation	PY1c: “Asked how the patient would like to be referred to. Restated what the patient reported to confirm accuracy. Warned patient about the experience of uncomfortable sensations throughout the various movements.” PY1k: “Explained what she was going to have him do and the reasoning behind it.” PY1bb: “She [PT] asked good questions. She did a good job learning about him [SP], how this affects his daily life and his goals.”
PY2	2.41 ± 1.43	Inquiry Explanation Screening	PY2a: “The PT explained all the steps well and ensured that the patient and her were on the same page throughout the examination.” PY2i:“She [PT] did a good job checking functional movement and mechanics with lifting. This gave her a good idea of what’s going on.” PY2k: “Screen for red flags, thorough exam questions, analysis of movements.” PY2u:” Screen red flags, ask about health history….”
PY3	2.63 ± 1.52	Interaction Inquiry Screening	PY3a: “She looked like she maintained good eye contact and sat at eye level with the patient during the interview.” PY3i: “She [PT] did a good job understanding the basics of the patient’s back pain--what makes it better, worse, when it started.” PY3o: “She[PT]asked appropriate questions to get a better understanding of the patient’s pain and examined multiple systems.” PY3v: “She asked further questions when the patient stated his stomach hurt from bending forward.”
PT	3.58 ± 1.70	Inquiry Screening Analysis Assessment	PT1: “They [PT] asked all pertinent questions in an open manner. They [PT] maintained eye contact throughout. They [PT] provided the patient with an outline of what to expect with the session including that they [PT] would take notes.” PT8: “Noticed abnormal response to movement and performed a fairly comprehensive screen. Performed a movement analysis.” PT8: “Captured most of the data needed to understand patients complete ICF (activities, participation, current health condition). PT7: “She [PT] developed good rapport with the patient, she asked pertinent questions, but did not dwell on the history taking portion of the exam…” PT11: “She [PT] was able to establish most of the SINSS (Missed irritability).” PT19: “… observed a functional movement that was a primary complaint for the patient, responded on her feet to new info about abdominal pain and was able to respond to appropriate screening questions.” PT26: “She [PT] screened potential red flag symptoms as the patient had abdominal pain…”

PY1, professional year 1, PY2, professional year 2, PY3, professional year 3, PT, licensed physical therapist.

Themes that emerged for potential improvement in the encounter included improving the subjective interview, refining the clinician-patient interaction, and the need for more thorough medical screening. First year (PY1) and second year (PY2) students identified 1.53 (±1.30) (1.63 ± 1.43) items, respectively, that the physical therapist could have done differently in the encounter with the SP. There was a noticeable increase in items and variation (2.96 ± 2.98) in 3rd year (PY3) students who have had more clinical experience through internships. Licensed physical therapists identified 3.69 (±2.56) items that the physical therapy could have done differently in the encounter (Table 5).

**Table 5:** Counts, themes and example quotes from narrative responses to what the reviewer identified the PT in the recording could have done better.

Level of experience	Item count for what the PT could have done better (Mean counts ± SD)	Themes	Example quote from narrative responses
PY 1	1.53 ± 1.30	Inquiry Explanation	PY1e: “I think she[PT]could have been better about her word choice in some scenarios, such as not apologizing when asking a patient to perform a needed test…” PY1h: “The PT asked several leading questions, and bounced around frequently.” PY1k: “Maybe explained a little more about what the tests are indicating after the results.” PY1aa: “Asked more about the pain the patient was feeling as they were doing the movements. Rate it, where is it, worse better same?”
PY2	1.63 ± 1.43	Inquiry Rapport with patient Examination items/sequence	PY2d: “Could have carried out conversation more after asking a question. Seemed to just go from one question to the next without having a conversation about an answer.” PY2i: “She [PT] could’ve checked passive ROM, accessory movement of lumbar spine and prone instability test.” PY2w: “Building a rapport with patient on personal experiences. Dig deeper into goals and activities that bring joy.” PY2bb: “The physical therapist should have created more rapport by making the patient comfortable, creating a relationship, and also doing more hands on examination tests.”
PY3	2.96 ± 2.98	Inquiry Examination items/sequence	PY3d: “I think that she should have asked the patient a few more questions regarding his abdominal pain, and performed an abdominal exam If she did not find anything, she could ask the patient to keep an eye on what he is eating for the next week to see if that does cause any pain changes.” PY3l: “I think the PT could have done a more thorough screen of the abdomen to rule out anything more sinister causing the patient’s back pain, especially after he mentioned the stomach pain.” PY3s: “I think they should have checked vitals after hx of HBP and w/ report of stomach pain.”
PT	3.69 ± 2.56	Sequencing Examination items Abdominal exam	PT1: “The ROM sequencing was interesting to me. I would have started with flexion and extension first and then moved towards side-bending and rotation.” PT2: “Given this patient’s age, gender and medical history I would have added a screen of patient vitals, and added some red flag screen questions to the subjective section of the exam.” PT8: “Capture a bit more information to understand the irritability and stability of the presentation (though could be discovered in objective exam). Captured a bit more information to understand the patients’ ICF (environment, personal factors)…” PT9: “I think if she started with symptom mapping it would have saved her a lot of time and helped tailor her subjective to be more focused, it wasn’t until her objective exam she realized that the patient had stomach pain as well (that may or may not be related) and she did not ask about any LE sx, numbness/tingling. This is crucial information to acquire.” PT25: “She[PT] could have done more palpation. She [PT] could have measured range of motion. She could have asked more questions of type of pain.” PT26: “I feel she [PT] could have explored the abdominal pain a little more in depth with potential palpation, further questioning.”

Four clinicians and two PY3 students participated in the interviews. Transcripts from the interviews were independently reviewed by the primary and secondary authors. The authors held discussions following their review and agreed on three main themes that materialized from the interviews: the importance of instructions prior to recording review, a review of a patient encounter allows for and enhances self-reflection, and the importance of a rubric or guide concurrently in reviewing video. Themes and exemplar quotes can be found in Table 6.

**Table 6:** Themes and example quotes from interviews.

Theme	Student or clinician	Example quote
Importance of instructions	PT5_7_2018	“I followed the PI’s [principal investigator’s] instructions and prompts.”
Review of encounter allow for self-reflection	PY3_03-1986	The recording “helped me see the flow and direction of subjective and how it focuses the next phases of the examination.”
Importance of a rubric or guide in reviewing video	PT7_19_2016	“I had a pen, paper and rubric to take notes.”

## Discussion

Results from this study indicate that the majority of participants found that the digital recording was a useful tool for reflecting on their professional ability and development. However, a variety of approaches in the review process were found across groups, especially between clinicians and students. Students across groups used “fast speed” and “fast forward,” while clinicians appeared more likely to take notes. In general, all groups described being focused throughout the digital recording review; clinicians appeared to have more consistent focus compared to students. These findings align with previous research which found benefits in the use of video recordings to reflect on medical interventions in a neonatal intensive care unit (24). Observing another clinician’s encounter with a patient allows the opportunity for objective comparison, and self-identification of clinical strengths and areas for improvement.

A secondary purpose of the study was to explore how review of a digital recording of a standardized patient encounter varied by level of student training, and between students and expert clinicians. There were increases in the number of observable items identified and the degree of analysis across levels of experience. The smaller numbers and depth of responses in the early (PY1 & PY2) groups are consistent with their level of knowledge and training. The complexity of student analysis increased with training. For example, PY1 and PY2 students focused more on the PT-SP interactions than clinical skills; PY3 students identified a general need for improvement in examination items; and clinicians provided specific suggestions for the PT’s approach, such as sequencing of the clinical examination and specific tests/measures to rule-in a condition. However, the fact that all levels of students and practicing clinicians identified elements of what the clinician performed well and could improve in the recording suggests that a generalized recording can be useful across multiple levels of training. These findings indicate a complex and technical approach by clinicians in the analysis of reviewing a recording.

It is important to recognize that prior to viewing the digital recording, participants in the study were instructed to consider two questions: “What did the PT do well?” and “What would YOU have done differently?” which are a modification of the Plus-Delta approach to debriefing simulated encounters (18, 19). Dzara et al. outline the importance of session alignment to meet educational goals as one component of incorporating videos in medical education (17). A necessary element of andragogical instructional design is for the educator to provide instructions and goals for viewing the video to serve as a scaffold for learning. Providing context and objectives is a necessity to frame the context for the learner/viewer.

The idea of learning through the observation of others has been around for decades (25). The use of video as an effective tool for the viewer to observe, learn, and reflect in medical education is not well described in the literature. Recent research by Weingartner and colleagues found that premedical students have improved clinical skills by observing video recordings of standardized patient encounters (26). The results of our study indicate that physical therapy students and physical therapists can self-reflect on their own clinical ability while observing another clinician’s encounter. This study supports the work by Salminen et al. who found that the use of virtual patients can facilitate self-reflection in medical students (6).

This study supports the use of a digital recording to promote student and clinician reflection. However, there are limitations to this study. The data were from a single-site convenience sample, which may not accurately reflect the general physical therapy community, which restricts generalizability. Further, no validated survey tool met the needs for assessment of viewing the recording. The lack of a validated survey instrument limits the reliability and consistency of the reported results. The study would have been enhanced by having more participants for interviews to gather additional perspectives and increase generalizability. Lastly, the use of blinded researchers to analyze qualitative responses from the survey and interviews would have strengthened the study by minimizing bias in data interpretation and increasing internal validity.

As online learning tools and instructional use of digital recordings becomes more prevalent; video review can be a useful low-cost learning modality. In conclusion, findings from this study provide evidence to support the use of video recordings to promote student and clinician reflection. This study demonstrates that review of a digital recording of a standardized patient encounter can be used facilitate reflection in practicing physical therapists. Future research would benefit from broader sampling and incorporation of standardized survey instruments. The development of best practices in designing the learning experience, including guidelines for student reflection and self-assessment across medical and health professions trainees would be valuable. Specifically, the use of an explicit framework to review their recordings, and the usefulness of that framework to enhance reflection-on-action and growth as a clinician, should be explored.

## Data Availability

The datasets for this study are not provided as the participants of this study did not give written consent for public sharing of their data.
