# Supplementary material for: Digital recordings of a clinical encounter facilitate reflection in physical therapy students and clinicians
Source: Front Med (Lausanne). 2025 Mar 11;11:1516724. doi: 10.3389/fmed.2024.1516724 (PMC11933041; doi:10.3389/fmed.2024.1516724)
Supplement: Supplementary file 1 [file Data_Sheet_1.docx]

**Appendix A**

Survey Questions

| **Demographics** |  |
| --- | --- |
| Question 1 | Please select your gender identification [Drop down response] |
| Question 2 | Please select your current level of clinical experience*   - PY1 (1^st^ year entry-level physical therapy student) - PY2 (2nd year entry-level physical therapy student) - PY3 (3rd year entry-level physical therapy student) - Resident - Fellow-in-Training - Currently a practicing physical therapist |
| Question 3 | Are you a licensed physical therapist [Yes/No] |
| Question 4 | To the nearest year, please enter your years of physical therapy experience [Fill-in] |
| Question 5 | Please specify your current practice setting [check all that apply]   - Inpatient hospital - Outpatient hospital - Home health - Private outpatient - Skilled-nursing facility - Long-term care - Other |
| Question 6 | Please indicate the following post-graduate training you have completed since finishing PT school [Check all that apply]   - ABPTRFE Board Certification - ABPTRFE Fellowship - Current certification program - Current resident or fellow - Graduate, physical therapy residency - Graduate, physical therapy fellowship - Non-residency/fellowship specialty certification (e.g., MTC, TPDN) - Post-graduate academic program (e.g., DSc, PhD) |
| Question 7 | Please indicate your perceived ability on the sliding scale [Sliding scale]  0: Beginner 50: Novice 100: Expert |
| **Assessment of Recording** |  |
| Question 8 | How would you rate the physical therapist’s performance in the recording? [Sliding scale]  0: Beginner 50: Novice 100: Expert |
| Question 9 | What did the physical therapist do well in the encounter? [Text box] |
| Question 10 | From your perspective, what do you think the physical therapist in the encounter should have done differently? [Text box] |
| Question 11 | If you were the physical therapist in the scenario and had 20 minutes remaining in the session, what would you do to manage this patient? |
| Question 12 | Based on the encounter in the recording, which of the following would you do next [Drop down: Treat, Treat & Refer, Refer] |
| Question 13 | If you selected “Treat & Refer” or “Refer” what is your concern? |
| **Process of Review** |  |
| Question 14 | **I remained entirely focused on the recording as I reviewed it** [Drop down: Strongly agree, Agree, Neutral, Disagree, Strongly disagree] |
| Question 15 | **At the time of reviewing the recording I would describe my mindset as:** [Drop down: Positive, Neutral, Negative] |
| Question 16 | **As I reviewed the recording, I performed the following [Click all that apply]**   - Fast forwarded - Played at fast speed - Paused - Reviewed other websites - Text messaged - Took notes - Listened to music - Watched television, movies or other videos - None of the above |
| Question 17 | **Please rate the level of agreement with the following statement:**  Review of this digital recording was a useful tool in reflecting on my own professional ability and development. [Drop down selection: Strongly agree, Agree, Neutral, Disagree, Strongly disagree] |

*=indicates “skip-logic” to question 6 if response was “PY1, PY2, PY3”
