# Supplementary material for: Digital recordings of a clinical encounter facilitate reflection in physical therapy students and clinicians
Source: Front Med (Lausanne). 2025 Mar 11;11:1516724. doi: 10.3389/fmed.2024.1516724 (PMC11933041; doi:10.3389/fmed.2024.1516724)
Supplement: Supplementary file 2 [file Data_Sheet_2.docx]

**Appendix B**

**Interview Questions**

**Opening Question**: Please share with us your current level of training and your current practice setting.

**Transition Question**: To the nearest day, when did you review the digital recording of the physical therapists’ encounter with the standardized patient?

**Key Question:** What preparations/steps/strategies did you take to review the recording?

**Probing Question (if necessary):** Did anyone have similar or different steps or strategies?

**Key Question:** Describe your degree of focus and engagement when reviewing the recording.

**Probing Question (if necessary):** Did you feel like you were solely focused on reviewing the recording? Did you have distractions?

**Key Question**: Was review of the recording of the physical therapists’ encounter a helpful tool in reflecting on your own professional abilities and development?

**Probing Question (if necessary):** Does anyone else feel the same way?

**Ending Question:** In the future, is there anything you would do differently to learn when reviewing a digital recording of a standardized patient encounter?

**Wrap-up:** Thank you for taking the time to participate in this focus group. Our session has ended.
