# Supplementary material for: Digital recordings of a clinical encounter facilitate reflection in physical therapy students and clinicians
Source: Front Med (Lausanne). 2025 Mar 11;11:1516724. doi: 10.3389/fmed.2024.1516724 (PMC11933041; doi:10.3389/fmed.2024.1516724)
Supplement: Supplementary file 3 [file Data_Sheet_3.docx]

**Appendix C**

**Regression Models-Ordinary Least Squares (OLS)**

1. Regression Model 1: Usefulness of digital recording review (variable: *ReviewUsefulTool(Numeric)*) was associated with year in training, controlling for demographic characteristics.

The fitted regression model was:

*ReviewUsefulTool (Numeric)=0.20PY1 +0.05PY2 +0.32PY3 +0.10male +2.88*

- Dependent Variable: ReviewUsefulTool(Numeric) is the numeric version of the 0-4 Likert scale response regarding the usefulness of the video tool.
- Dummy Variables: PY1, PY2, and PY3 indicating membership in a specific year of PT school. Reference group for these PY variables is licensed PT.
- Additional dummy variable: male is a dummy variable coded as “1” for males and “0” for females and anyone else not identifying as a male.

Note: Non-males included those that identified as agender and non-binary. They were combined with females and treated as members of the majority group. There were not enough people in the agender and non-binary categories to analyze separately

1. Regression Model 2: Level of experience of the reviewer was associated with level of Review Focus (Numeric).

The fitted regression model was:

*ReviewFocus (Numeric) = -0.45PY1+-0.66PY2+-0.36PY3 +3.32*

- Dependent Variable: ReviewFocus(Numeric) is the numeric version of the 0-4 Likert scale response regarding the usefulness of the video tool.
- All other variables were coded identically as the Regression model 1.
